# Poly(ADP-Ribose) Polymerases in Plants and Their Human Counterparts: Parallels and Peculiarities

**DOI:** 10.3390/ijms20071638

**Published:** 2019-04-02

**Authors:** Dagmar Rissel, Edgar Peiter

**Affiliations:** 1 Plant Nutrition Laboratory, Institute of Agricultural and Nutritional Sciences, Faculty of Natural Sciences III, Martin Luther University Halle-Wittenberg, 06099 Halle (Saale), Germany; 2 Agrochemisches Institut Piesteritz e.V. (AIP), Möllensdorfer Strasse 13, 06886 Lutherstadt Wittenberg, Germany; 3 Institute for Plant Protection in Field Crops and Grassland, Julius Kühn-Institut (JKI), 38104 Braunschweig, Germany

**Keywords:** abiotic stress, *Arabidopsis thaliana*, PAMP, PARP, poly(ADP-ribose) polymerase, poly(ADP-ribosyl)ation, SRO protein

## Abstract

Poly(ADP-ribosyl)ation is a rapid and transient post-translational protein modification that was described first in mammalian cells. Activated by the sensing of DNA strand breaks, poly(ADP-ribose)polymerase1 (PARP1) transfers ADP-ribose units onto itself and other target proteins using NAD^+^ as a substrate. Subsequently, DNA damage responses and other cellular responses are initiated. In plants, poly(ADP-ribose) polymerases (PARPs) have also been implicated in responses to DNA damage. The Arabidopsis genome contains three canonical PARP genes, the nomenclature of which has been uncoordinated in the past. Albeit assumptions concerning the function and roles of PARP proteins in planta have often been inferred from homology and structural conservation between plant PARPs and their mammalian counterparts, plant-specific roles have become apparent. In particular, PARPs have been linked to stress responses of plants. A negative role under abiotic stress has been inferred from studies in which a genetic or, more commonly, pharmacological inhibition of PARP activity improved the performance of stressed plants; in response to pathogen-associated molecular patterns, a positive role has been suggested. However, reports have been inconsistent, and the effects of PARP inhibitors appear to be more robust than the genetic abolition of *PARP* gene expression, indicating the presence of alternative targets of those drugs. Collectively, recent evidence suggests a conditionality of stress-related phenotypes of *parp* mutants and calls for a reconsideration of PARP inhibitor studies on plants. This review critically summarizes our current understanding of poly(ADP-ribosylation) and PARP proteins in plants, highlighting similarities and differences to human PARPs, areas of controversy, and requirements for future studies.

## 1. Introduction

**Figure 1:**
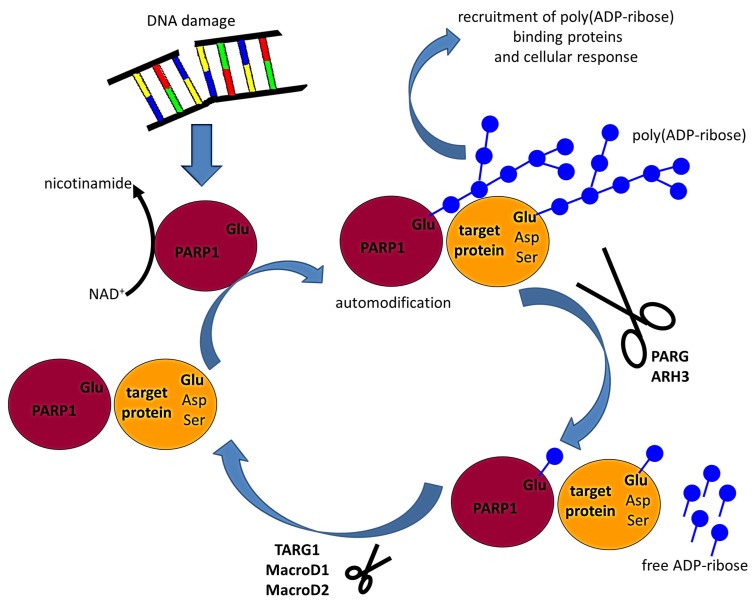
Cellular ‘life cycle’ of ADP-ribose polymers as inferred from studies on the human proteins. Activated upon DNA strand break, poly(ADP-ribose) polymerase1 (PARP1) and PARP2 catalyze the transfer of ADP-ribose molecules onto itself and other target proteins. The generated ADP-ribose polymers serve as scaffolds recruiting proteins containing various poly(ADP-ribose)-binding domains, which initiates cellular responses. The ribose-ribose bonds are hydrolyzed by Poly(ADP-ribose) glycohydrolase (PARG), ADP-ribose hydrolase 3 (ARH3), Terminal ADP-ribose glycohydrolase 1 (TARG1), MacroD1, and MacroD2, allowing rapid poly(ADP-ribose) turnover and controlled cellular signaling processes.

Poly(ADP-ribosyl)ation describes the rapid and transient posttranslational transfer of negatively charged ADP-ribose molecules onto proteins. First, ADP-ribose moieties are covalently attached to the target proteins. Subsequently, poly(ADP-ribose) chains of various length and branching complexity are synthesized, forming O-glycosidic bonds between the ADP-ribose molecules [1,2,3,4,5] (Figure 1). The enzymes catalyzing poly(ADP-ribosyl)ation are named poly(ADP-ribose) polymerases (PARPs). NAD^+^ serves as substrate for poly(ADP-ribose) synthesis, and nicotinamide is formed as a concomitant product [3,6,7]. The synthesized ADP-ribose polymers constitute an interaction platform to modulate cellular responses. In animals, several poly(ADP-ribosyl)ation site-containing proteins and poly(ADP-ribose) binding motif-containing proteins have been identified acting as poly(ADP-ribose) readers translating the poly(ADP-ribose) signal into cellular responses [8,9]. These proteins are components of essential cellular processes such as DNA repair, cell cycle checkpoints, chromatin remodeling, signaling, protein degradation, and cell death [10,11,12,13,14,15,16,17,18,19,20,21,22,23,24,25]. The amino acids modified by poly(ADP-ribosyl)ation in the target proteins are predominantly glutamic acid and aspartic acid; modification is performed via ester linkage [26,27,28]. Recently, the modification of serine residues of target proteins by O-glycosidic bonds has been shown [29]; the enzymatic modification of lysine residues is currently a matter of debate [27,28].

In contrast to their mammalian counterparts, less is known about the functions of plant poly(ADP-ribose)polymerases. In this review, we will first briefly highlight the current understanding of the function and roles of PARP proteins in humans and subsequently elaborate the similarities and differences in structure and function of their plant homologs. We will focus on plant-specific roles, such as in seeds, and in the determination of stress resistance by those proteins, which is currently a matter of controversy.

## 2. Poly(ADP-Ribosyl)ation and Poly(ADP-Ribose) Polymerases (PARPs) in Humans

### 2.1. PARPs Constitute a Heterogeneous Protein Family in Humans

PARP proteins have been found in all kingdoms of life except yeast [30,31]. In the human genome, 17 PARP genes have been identified [5,32,33,34]. They constitute a heterogeneous protein family with distinct structural domains, subcellular localizations, activities, and functions [32]. According to their structures and functions, the different PARP proteins were classified as DNA-dependent PARPs (PARP1, PARP2, PARP3) which are activated upon DNA damage; Tankyrases (PARP5a, PARP5b) which are involved in telomere homeostasis, DNA repair, mitotic spindle formation, and cellular signaling; Cys-Cys-Cys-His zinc finger and WWE poly(ADP-ribose)-binding domain-containing PARPs (PARP7, PARP12, PARP13.1, PARP13.2); and poly(ADP-ribose)-binding macrodomain-containing PARPs (PARP9, PARP14, PARP15) [8,27,32,35]. The last two groups are defined according to their protein structure, and, so far, little is known about the function of their members. The remaining PARP proteins are grouped as unclassified PARPs, as their domain architecture differs from each other and from the other groups [35]. To catalyze poly(ADP-ribosyl)ation, the catalytic triad motif H-Y-E within the catalytic PARP domain is essential but not sufficient [36,37]. Therefore, only PARP1, PARP2, and the Tankyrases are bona fide PARPs. PARP3, PARP4, PARP6, PARP10, PARP14, PARP15, and PARP16 were found to exhibit mono(ADP-ribosyl)ation activity catalyzing the addition of a single ADP-ribose molecule onto target proteins. No catalytic activity has been found for PARP9 and PARP13 [37].

### 2.2. PARP1, PARP2, and PARP3 Are Activated upon DNA Damage

**Figure 2:**
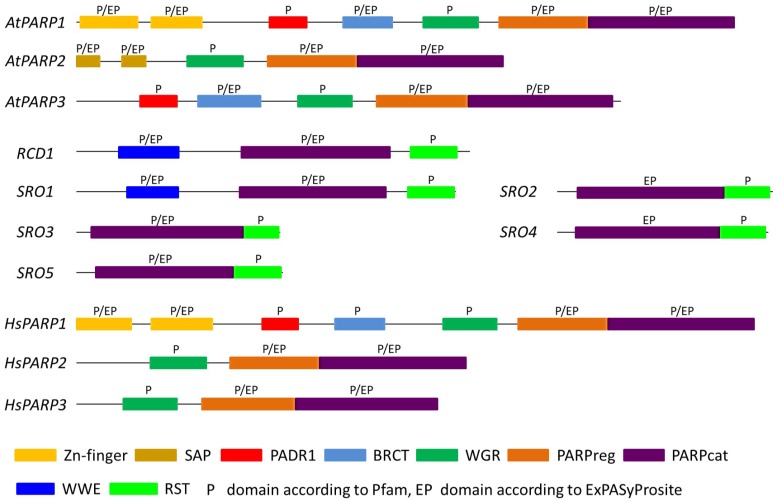
Schematic representation of domains in PARP proteins from humans and *Arabidopsis thaliana*. Domains were defined according to Pfam 27.0 and are displayed as colored boxes. ExPASY Prosite indicated the existence of PARPcat domains also in SRO2 and SRO4 which are absent in the Pfam analysis. Figure taken from [47].

The best-studied PARP protein is the founding member of the protein family, PARP1 [5]. It is the most abundant PARP enzyme in mammalian cells and accounts for approximately 85% of poly(ADP-ribosyl)ation activity [32]. Human PARP1 is a 113 kDa protein with a well-defined modular architecture (Figure 2) [32]. It possesses an N-terminal DNA interaction domain, a central automodification domain, and a C-terminal catalytic domain [9,32,38]. This catalytic region is highly conserved in mammals, particularly the 50 amino acid-spanning so-called “PARP signature” [38]. The PARP signature forms the active site of the PARP proteins and exhibits 100% conservation among vertebrates and 92% among all species [38]. Additionally, a WGR domain, named after its repeating amino acid motif (W-G-R), is located in the catalytic region. Apart from the glutamate moieties that allow automodification of PARP1, the central automodification domain contains a BRCT (breast cancer susceptibility gene 1 C-terminus) domain that is known to be involved in protein–protein interactions [39]. This domain is commonly found in DNA damage response proteins [39]. Three zinc fingers, a bipartite nuclear localization signal, and a caspase (cysteine proteases cleaving at an aspartic acid) cleavage site form the DNA-binding domain. The two homologous zinc fingers Zn1 and Zn2 are able to bind to DNA single and double strand breaks and abnormal DNA structures [40,41,42]. The third zinc finger, Zn3, is structurally unique and required for the activation of DNA-dependent catalytic activity of PARP1 [43]. Upon association with damaged DNA, the Zn3 and WGR domains refold to allow enhanced interdomain contacts and facilitate PARP1 catalytic activity [42]. Additional conformational changes within PARP1 lead to further activation [8,44,45,46].

Upon activation, PARP1 automodification and poly(ADP-ribosyl)ation of target proteins take place, recruiting DNA damage response proteins to the lesions (Figure 1). The negative charges of automodified PARP1 proteins repulse proteins from the DNA to allow access for the DNA damage repair machinery [9]. PARP1 has been found to be involved in virtually all DNA damage response pathways. Upon sensing DNA single strand breaks, PARP1 automodification recruits XRCC1 which scaffolds the assembly and activation of the base excision repair machinery and the subsequent repair of the small lesions caused by oxidation or alkylation [3,48,49,50]. Additionally, PARP1 was implied to be involved in homologous recombination (HR) repair of DNA double strand breaks (DSB), since components of the HR machinery such as MRE11 (mitotic recombination 11) and ATM (ataxia telangiectasia-mutated) are rapidly recruited to DNA damage sites in a poly(ADP-ribose)-dependent manner [13,14,51]. In line with this, PARP1 acts as a facilitator of HR repair at stalled DNA replication forks, as PARP1 binding to the stalled fork prevents the assembly of the non-homologous end joining (NHEJ) complex [9,52,53]. Contrastingly, PARP1 was also shown to interact with Ku70/Ku80 proteins, which are known key players in NHEJ [53,54]. However, its precise role in NHEJ is intricate, as classical NHEJ still proceeds in the absence of PARP1 [53]. As PARP1 competes with Ku proteins for DNA binding, it appears not to be a core component of classical NHEJ but of an alternative NHEJ pathway [55]. In line with its function in DNA damage responses, PARP1 also acts in chromosome remodeling, establishment and maintenance of heterochromatin, and transcriptional regulation [4,8,25,56]. In addition, PARP1 has also been implicated in various processes of cell death, such as apoptosis, parthanatos, programmed necrosis, and autophagy [24]. The common denominator of all these processes is an excessive formation of poly(ADP-ribose). During apoptosis, the increased PARP1 activity depletes the cellular energy pool, leading to an activation of caspases. The caspase-mediated cleavage of PARP1 is a hallmark of apoptosis, leading to the accomplishment of apoptotic cell death. Upon excessive activation of PARP1, poly(ADP-ribose) molecules appear to leave the nucleus and invade the mitochondria. Here, poly(ADP-ribose) is bound by AIF (apoptosis-inducing factor), which in turn is translocated to the nucleus where it promotes DNA fragmentation leading to parthanatos. During programmed necrosis and autophagy, PARP1 interacts with key components of both pathways, hence promoting cell death [24].

PARP2 was identified in a study initiated to resolve the source of residual poly(ADP-ribose) formation in cells lacking PARP1 [57]. It accounts for approximately 15% of overall cellular poly(ADP-ribose) synthesis [32]. The domain architecture of PARP2 differs from that of PARP1 (Figure 2). PARP2 consists of an N-terminal region (NTR) containing a nuclear and a nucleolar localization signal and a caspase cleavage site, a WGR domain, and a catalytic PARP domain [57,58,59]. The NTR lacks the zinc finger domain found in PARP1 and is an intrinsically disordered protein region allowing flexible adaptation to various damaged DNA structures such as gaps, flaps, and recombination intermediates [59]. However, the NTR does not localize to DNA damage sites on its own. The WGR and the catalytic domain are necessary and sufficient to direct PARP2 to DNA damage sites. The WGR domain is also involved in the activation of poly(ADP-ribosyl)ation by PARP2 [59]. The catalytic domains of PARP1 and PARP2 exhibit 69% homology [57]. Additionally, the catalytic domain was found to contribute to the binding affinity and the localization to the DNA damage sites [59]. Even though PARP2 lacks a conserved automodification domain, it is capable of automodification [58]. PARP1 and PARP2 were found to form heterodimers and poly(ADP-ribosyl)ate each other [60]. Additionally, PARP2 has been shown to interact with an overlapping, but distinct, set of proteins compared to PARP1 [61]. In line with this, PARP2 was found to be an actor in the response to DNA single strand break, similar to PARP1 [60,62]. Moreover, redundancy between PARP1 and PARP2 has been shown in cell survival and at stalled replication forks [63,64]. PARP2 has also been found to be involved in the choice of DSB repair modes, channeling repair towards HR or an alternative NHEJ [65]. The exact roles of PARP1 and PARP2 in DNA DSB response still have to be further elucidated. 

To date, less information is available on PARP3. Similar to PARP1 and PARP2, it is activated by DNA strand breaks [4] and it confers mono(ADP-ribosyl)ation activity [32] and functions in DNA DSB repair [66,67]. Thereby, PARP3 is efficiently recruited to DNA damage sites and interacts with proteins of the classical NHEJ pathway [66,68]. Additionally, PARP3 prevents DNA end resection, and as a consequence promotes classical NHEJ [53]. Apart from this, PARP3 mono(ADP-ribosyl)ates PARP1, kick-starting its activation [69].

### 2.3. The Removal of Poly(ADP-Ribose) Is a Two-Step Process

So far, five enzymes have been identified to degrade poly(ADP-ribose) chains, allowing rapid and dynamic poly(ADP-ribose) turnover (Figure 1) [8,9]. The key enzyme among them is poly(ADP-ribose)glycohydrolase (PARG) [30,70,71]. PARG possesses exoglycosidic and endoglycosidic activity, hydrolyzing terminal and internal ribose-ribose linkages, respectively, hence releasing ADP-ribose oligomers [71,72,73]. PARG enzymes, however, are not capable of cleaving the ester bond between the ADP-ribose molecule and the acceptor amino acids of the target protein. To date, only one PARG gene has been identified in mammals, but five protein isoforms resulting from alternative splicing have been found in different cellular compartments [74,75]. PARG protein function is essential, as genetic deletions of PARG in mice or *Drosophila* are lethal [76,77]. Similar to PARG, ADP-ribosyl-hydrolase 3 (ARH3) was found to exhibit poly(ADP-ribose)-hydrolyzing activity in the nucleus, the cytosol and the mitochondrion (Figure 1) [78,79]. ARH3 shares only little structural similarity with PARG; it accounts for 10% of the poly(ADP-ribose)-hydrolyzing activity in the cell [78,79]. The macrodomain-containing proteins Terminal ADP-Ribose protein Glycohydrolase 1 (TARG1) and Macrodomain-containing protein D1 (MacroD1) and MacroD2 possess the ability to hydrolyze the ester bond between the ribose and the acceptor amino acid (Figure 1) [80,81,82].

## 3. Poly(ADP-Ribosyl)ation in Plants

### 3.1. Three Canonical PARP Proteins Have Been Identified in the Model Plant Arabidopsis thaliana

In the late 1970s, poly(ADP-ribosyl)ation activity was shown in higher plants by the incorporation of [^3^H]NAD into nuclei of onion and wheat embryo cells and onion meristematic root tissues [83,84,85,86]. This incorporation was found to be an enzymatic reaction covalently linking poly(ADP-ribose) molecules to carboxyl groups of the target proteins [87]. Lysine-rich histones H1, H2A and H2B, but not arginine-rich histones H3 and H4 were identified as acceptor proteins for poly(ADP-ribose) molecules [86,87]. In addition, automodification of a 114 to 116 kDa protein was described in these early times of poly(ADP-ribose) research in plants [87,88]. 

The first *PARP* gene identified in plants was *Arabidopsis thaliana APP* (At4g02390) [89]. In this review, APP will be called AtPARP2 as it is structurally most similar to human PARP2 (Figure 2; Table 1). The *AtPARP2* cDNA was identified due to its 62% similarity to the catalytic domain of human PARP1 during experiments carried out to identify *Arabidopsis* proteins that allow yeast cells to grow under stress conditions. The AtPARP2 protein consists of 637 amino acids and has a size of 72 kDa. The PARP signature is conserved in AtPARP2. Apart from that, a nuclear localization signal and an automodification domain were found. In contrast to human PARP1, which possesses N-terminal zinc-finger domains, AtPARP2 contains an N-terminal SAP domain (Figure 2). The SAP domain is a putative DNA-binding domain involved in nucleic acid metabolism, named after three proteins that contain it (SAF-A/B, Acinus and PIAS) [90]. Expression of *AtPARP2* in yeast revealed a nuclear localization and poly(ADP-ribosyl)ating activity. The main polymer size was 10 to 15 residues, but polymers of up to 40 ADP-ribosyl residues were formed [91]. The poly(ADP-ribosyl)ating activity was reduced by PARP inhibitors, 3-aminobenzamide (3AB) and nicotinamide. Nuclear localization of AtPARP2 in planta has been confirmed by transient expression of AtPARP2-GFP constructs in *Nicotiana benthamiana* and *Arabidopsis* [92,93,94]. It has recently been shown that nuclear import of AtPARP2 is mediated by Importin-α [94]. In addition to its nuclear localization, AtPARP2 has been suggested to be partially localized in chloroplasts [93]. Promoter-GUS fusions and RNA in situ hybridization studies showed *AtPARP2* expression in imbibed seeds, the vegetative meristem of the shoot apex, stamen of open flowers, and late stages of embryo development [93].

*AtPARP1* (At2g31320) was identified in a screen for ionizing radiation-induced genes in *Arabidopsis thaliana* [95]. AtPARP1 consists of 983 amino acids and exhibits conserved structural motifs compared to human PARP1. Similar to human PARP1, AtPARP1 contains a conserved catalytic domain, zinc finger motifs, and a nuclear localization motif (Figure 2). The central automodification domain is less conserved, but glutamate residues are present, allowing auto poly(ADP-ribosyl)ation. Apart from this, the BRCT domain, allowing protein–protein interactions, is also less conserved. Generally, the structural similarities between AtPARP1 and human PARP1 indicate functional similarities. This assumption was recently further supported by a yeast expression study, in which *AtPARP1* expression inhibited yeast cell growth similarly to *HsPARP1* [96]. Growth inhibition by both proteins was reverted by the addition of the PARP inhibitors 3AB and 6-(5H)-phenanthridinone [96].

Like AtPARP2, AtPARP1-GFP was found to localize to the nucleus in *Nicotiana benthamiana* and in *Arabidopsis* cell suspension culture [92,93]. Additionally, PARP1-GFP was detected in chloroplasts and mitochondria when expressed in *Arabidopsis* protoplasts [93]. Fusion of the putative *AtPARP1* promoter with *GUS* and RNA hybridization analyses revealed expression of *AtPARP1* in the roots, apices of inflorescences, vegetative meristem, and during the late stages of embryo development [93].

Unfortunately, the nomenclature of *Arabidopsis PARP1* and *PARP2* genes and proteins has been inconsistent in the literature (Table 1). In this review, the following nomenclature is used, which we recently suggested [47]: *AtPARP1* refers to the gene At2g31320, and *AtPARP2* refers to At4g02390. The nomenclature of mutants of these genes has been likewise inconsistent and redundant. For a unified nomenclature of published mutants, the reader is referred to [47].

So far, less is known about *AtPARP3* (At5g22470), which was first identified by *Arabidopsis* genome analysis. It was mainly expressed in seeds, but also in seedlings and roots of adult plants [93,101,109,110]. In addition, expression of *AtPARP3* was strongly induced (albeit at a low absolute level) in leaves by severe abiotic stress in the form of paraquat, NaCl, high light, or desiccation [104]. PARP3-GFP fusions localized to the nucleus [93,110], and in one study also to the cytosol [93]. 

In contrast to the expression of *AtPARP1* in yeast cells, *AtPARP2* inhibited yeast growth only partially, while *AtPARP3* expression did not affect growth [96]. This may be attributed to differences in or lack of the DNA-binding domain in AtPARP2 and AtPARP3. The DNA-binding domain of HsPARP2 is also structurally different from that of HsPARP1, but *HsPARP2* expression causes an inhibition of yeast growth [111]. The differential growth inhibition of yeast indicated that *Arabidopsis* and human PARPs share structural or functional features, but also pointed to plant-specific functions of AtPARP2 and AtPARP3.

### 3.2. In Contrast to Mammals, Plants Possess More Than One Poly(ADP-ribose) Glycohydrolase (PARG) Gene

In total, three genes with homology to human *PARG* have been identified in *Arabidopsis thaliana* [109]. For one of them, no expressed sequence tags (ESTs) or cDNA has been found so far. Therefore, this gene is classified as a pseudo-gene. The other two genes, *AtPARG1* (At2g31870) and *AtPARG2* (At2g31865), are localized in tandem on chromosome 2.

In *Arabidopsis*, both PARG proteins are localized in the nucleus, the cytoplasm, and at the plasma membrane [101,108]. AtPARG1 (also known as TEJ) was first identified in a genetic screen for altered circadian period length in *Arabidopsis* [112]. Mutant plants carrying a G262E substitution in the AtPARG1 protein showed a prolonged free-running period regarding the expression of circadian clock-controlled genes and leaf movement. These *tej* mutants also flowered earlier. These phenotypes suggest a general clock defect, making AtPARG1 a component of clock function in plants. Poly(ADP-ribose) polymer levels were increased in the *tej* mutants suggesting that AtPARG1 is a bona fide PARG [112]. Poly(ADP-ribose)glycohydrolase activity of AtPARG1 has been validated in vitro and in vivo [101]. Western blot and autoradiography of ^32^P-NAD^+^ revealed that recombinant AtPARG1 is able to remove poly(ADP-ribose) from automodified AtPARP2. Similarly, co-expression of *AtPARP2* and *AtPARG1* in *Arabidopsis* protoplasts led to a significant removal of poly(ADP-ribose) from automodified AtPARP2. In contrast to this, no poly(ADP-ribose)glycohydrolase activity towards automodified AtPARP2 or AtPARP1 was found for AtPARG2 in vitro and in vivo. This lack of activity could not be exclusively attributed to the presence of a polymorphism in the conserved PARG signature motif in PARG2, since a recombinant PARG2 protein carrying the conserved PARG motif did also not show any detectable poly(ADP-ribose) glycohydrolase activity. Therefore, additional deviations in the protein sequence of AtPARG1 and AtPARG2 are thought to account for the differences in enzyme activity [101].

Proteins that possess the ability to hydrolyze the ester bond between the ribose and the acceptor amino acid in Arabidopsis have not been determined yet. However, the proteins encoded by the loci At1g63410, At1g69340, and At2g37710 show considerable homology to the human MacroD1 and MacroD2 proteins described above and are thus candidates for this function. There seem to exist no *Arabidopsis* proteins homologous to human ARH3 and TARG1.

### 3.3. Plant PARPs Play a Role in DNA Damage Response and Genome Integrity

During about 40 years of work on poly(ADP-ribosyl)ation in plants, various studies showed that plant PARP proteins are components of DNA damage responses similar to their mammalian counterparts. The expression of *AtPARP1* and *AtPARP2*, but not *AtPARP3*, is induced by treatment with DNA-damaging agents, such as ionizing radiation, zeocin (a radiomimetric drug that induces DSB) or cisplatin (an inhibitor of DNA replication by cross-linking neighboring guanine bases) in *Arabidopsis* [95,97,113]. *AtPARP3* expression was only induced in the absence of *AtPARP1* or *AtPARP2* [97]. In contrast, *HvPARP3* expression was induced in barley roots in response to bleomycin (a glycopeptide that mainly induces DSB) [114]. Similar to *AtPARP* expression, PARP activity was induced by DNA-damaging agents, such as zeocin and X-ray irradiation [115,116]. Recombinant AtPARP1 and AtPARP2 are activated by nicked DNA as shown by automodification of the recombinant AtPARP proteins [91,95,101,116]. For AtPARP2, automodification was also shown in vivo [101]. Automodification of proteins in vitro and in vivo was blocked by the addition of the pharmacological PARP inhibitor 3AB [91,116].

In agreement with increased gene expression and activity, the genetic inhibition of AtPARPs in *parp1* and *parp2* mutant plants enhanced the sensitivity of plant growth to methyl methane sulfonate (MMS, a DNA alkylation agent that induces N-alkyl lesions and single strand breaks (SSB)) and to bleomycin [97,100,108,117]. Similarly, formation of true leaves was reduced in *parp2* seedlings grown on bleomycin and mitomycin C (a DNA cross-linking agent) [92]. Some authors reported that *parp2* mutants are more sensitive to DNA-damaging agents than *parp1* mutants [92,97]. In line with this, poly(ADP-ribosyl)ation was strongly reduced in *parp2*, but not in *parp1* mutants [92]. Increased plant damage was observed in *parp1 parp2* double mutants compared to the corresponding single mutants, indicating that both *AtPARP* genes are involved in responses to DNA-damaging agents [92,97,100,108]. This notion is further supported by an enhanced expression of *AtPARP1* in *parp2* mutants and vice versa [47,92,97]. Additionally, AtPARP1 and AtPARP2 were shown to physically interact with each other [92,116]. No exacerbation in the severity of plant damage was observed in *parp1 parp2 parp3* triple mutants, indicating that AtPARP3 is not active in DNA damage response in seedlings [108]. However, in barley, *HvPARP3* mutation led to an altered root growth response to bleomycin [114]. In summary, plant damage and reduced growth of *parp* mutants under genotoxic stress may be explained by aggravated DNA damage. 

In addition to DNA-damaging agents, infection of plants with the bacterium *Pseudomonas syringae* pv. tomato (*Pst*) was shown to induce DNA damage [118]. In line with this, DNA damage was enhanced in *parp2* and *parp1 parp2* mutants in response to treatment with *Pst* [92].

#### 3.3.1. PARP1 and PARP2 Are Actors in Various DNA Damage Response Pathways in Plants

The exacerbated sensitivity of *Arabidopsis parp* mutants to various DNA-damaging agents showed that plant PARPs are important actors in DNA damage responses, similar to their human counterparts. In line with this, the ionizing radiation-mediated induction of *AtPARP2* was found to depend on the presence of ATM, as the induction was absent in *atm* mutant plants [119]. ATM is an initiator of various DNA damage repair pathways [120] (see Section 2.2). Yet, the involvement of plant PARP proteins in specific DNA repair pathways still has to be elucidated. For instance, the PARP inhibitor 3-methoxybenzamide (3MB) increased the number of recombination events in Arabidopsis and tobacco [121]. This indicates that PARPs negatively regulate DNA repair by HR. In line with this, expression of the HR components *AtRAD51* and *AtXRCC3* was enhanced by 3AB treatment [122]. Moreover, expression of *AtPARP2* was induced in *mms21-1* mutant plants, while homologous recombination events were found to be reduced in this mutant [113]. *AtMMS21* encodes a SUMO E3 ligase, a critical component of the SMC5/6 complex which fulfills a central role in genome stability maintenance [113]. In contrast, expression of *AtXRCC2*, another component of HR, was found to be reduced upon 3AB treatment [97,122]. These apparently contradicting findings still have to be elucidated. 

Another study indicated that PARPs are involved in an error-prone alternative pathway of NHEJ like their mammalian counterparts [100]. Triple mutant plants lacking AtPARP1, AtPARP2, and AtKu80, a component of classical NHEJ, were more sensitive to MMS than *parp1 parp2* and *ku80* mutants, indicating that different DNA damage response pathways are impaired in the mutants [100]. Additionally, the extent of DNA damage was higher in the *parp1 parp2 ku80* mutant plants. A cell-free end-joining assay revealed a higher number of large deletions (>10 bp) at the ends of broken DNA strands in *ku80* and *parp1 parp2 ku80* than in *parp1 parp2* mutants. So, resection of nucleotides from the DNA ends occurred mainly in the *ku80* and *parp1 parp2 ku80* mutants. An alternative NHEJ pathway in *ku80* mutants is microhomology-mediated end-joining (MMEJ). A higher level of MMEJ products were found in *ku* mutants compared to *parp1 parp2* or *parp1 parp2 ku80* mutants, indicating that PARPs are involved in MMEJ [100]. In contrast, Shen and colleagues suggested that PARPs act in alternative NHEJ-independent of micro-homology [123].

Recently, one function of AtPARP1 in DNA damage responses was further clarified. A *parp1 rad5a* double mutant was more sensitive to the DNA-alkylating agent MMS than the corresponding single mutant plants [117]. By contrast, *parp1 rad5a* did not display enhanced sensitivity to the crosslinking agents cisplatin and mitomycin C as compared to the *rad5a* mutant plants. No enhanced sensitivity to either agent was observed in the *parp1* mutant compared to wild type plants. These findings suggested that AtPARP1 is involved in the repair of base alkylations in a pathway parallel to that involving RAD5a, which possibly corresponds to base excision repair (BER) and HR-independent single strand break repair [117]. In line with this, 3AB repressed paraquat-induced *XRCC1* expression [122]. XRCC1 is a component of the gap-filling and nick-sealing step of BER [124]. *AtPARP2* expression is induced in the absence of Ligase1, another component of BER, indicating that AtPARP2 also interacts with the BER pathway [91]. 

#### 3.3.2. PARP3 Is a Core Component of Genome Integrity in Seeds

In adult *Arabidopsis* plants, AtPARP3 was suggested to be either inactive or not involved in DNA damage responses [108]. In contrast, strong expression was found in seeds [101,109]. A screening of publicly available microarray datasets and histochemical GUS analyses confirmed the accumulation of *AtPARP3* transcripts specifically in the embryo and the endosperm of dry seeds, during imbibition, and during seed germination [110]. A similar expression pattern was also found for the *PARP3* orthologue in rice [125]. In addition, poly(ADP-ribose) levels did not correlate with the depth of seed dormancy in different ecotypes of Arabidopsis, but with their sensitivity to MMS [126,127]. Hence, AtPARP3 was suggested to be involved in protecting the plant embryo from DNA damage in the seed. This notion is supported by the findings that *AtPARP3* promoter activity coincided with reactive oxygen species (ROS) accumulation in the embryo and that *parp3* mutant seeds lose viability upon prolonged storage or upon artificial ageing, pointing to a role of AtPARP3 in ROS-induced DNA damage responses during seed storage and germination [110,128]. Database searches reveal the presence of sequences homologous to *AtPARP3* in other plant species, such as *Populus trichocarpa*, *Physcomitrella patens*, *Oryza sativa*, *Brachypodium distachyon*, *Sorghum bicolor*, *Zea mays*, and *Hordeum vulgare* [110,114,125]. This leads to the conclusion that the protective role of PARP3 may be conserved throughout the plant kingdom. 

The activity of PARPs produces the feedback inhibitor nicotinamide as byproduct [7]. Nicotinamidases convert nicotinamide to nicotinate, which is further metabolized back to NAD^+^ in the salvage pathway [7]. In *Arabidopsis*, the expression pattern of *AtPARP3* correlates with that of *Nicotinamidase 2* (*NIC2*) [126]. Germination of *nic2* mutant seeds was hypersensitive to MMS, potentially due to reduced levels of poly(ADP-ribosyl)ation, which are, in turn, likely to be due to a reduced nicotinamide degradation [126,127]. On the other hand, overaccumulation of nicotinate from nicotinamide degradation is toxic. Consequently, nicotinate detoxification by O-glycosylation, mediated by the nicotinate glycosyltransferase UGT74F2-1, has been shown to be required for seed germination under stress [129]. This is further evidence for the induction of PARP3 activity in seeds under unfavorable conditions.

Since AtPARP3 shows structural homologies to AtPARP1 and human PARP3 (Figure 2), similar functions may be assumed. As discussed above (Section 2.2), human PARP3 is involved in non-homologous end-join (NHEJ) repair of DNA damage, suggesting a role of plant PARP3 in DSB repair via NHEJ. Interestingly, in *Hordeum vulgare*, expression of *HvPARP3* in young seedling roots was enhanced by the DNA DSB-inducing agent bleomycin [114], supporting such a function. Yet, the exact role of plant PARP3 in DNA DSB repair has to be elucidated. Besides germination of Arabidopsis *parp3* mutant seeds in the presence of DNA DSB-inducing agents, crossing of *parp3* mutants with *ku70* and *ku80* mutants may provide evidence whether AtPARP3 acts in NHEJ like its mammalian counterpart. Since direct evidence of poly(ADP-ribosyl)ation activity of AtPARP3 is lacking so far, PARP activity needs to be determined in *parp3* mutant seeds. Aberrant poly(ADP-ribose) levels will indicate whether AtPARP3 is capable of poly(ADP-ribosyl)ation. However, a mono(ADP-ribosyl)ation activity by this enzyme, similar to its human counterpart, is also conceivable. 

#### 3.3.3. Like PARPs, Plant PARG Proteins Are Linked to DNA Damage Response

Similar to *parp* mutant plants, Arabidopsis *parg1* mutants exhibited enhanced sensitivity to mitomycin C and bleomycin [130]. Mutant plants lacking AtPARG2 displayed no or only slightly increased sensitivity to DNA-damaging agents, indicating that AtPARG1 is more important in the response to DNA damage caused by DNA-damaging agents [108,130]. Notably, *parg1* mutations induced more severe plant damage than the lack of AtPARPs [108]. This was attributed to the fact that free poly(ADP-ribose) is thought to be toxic to plant cells, as it has been described for mammalian cells [108].

The mechanistic role of PARGs in DNA damage responses of plants still has to be elucidated. Enhanced expression of HR components (i.e., *SMC6A*, *SMC6B*, *RAD17*, *RAD51*, *RAD54*, *REV7*) and NHEJ components (i.e., *LIG4*, *Ku70*, *Ku80*) was found in *parg1* mutants [108]. In contrast, *RAD51* and *SWI* expression induced by the bacterial elicitor flg22 were disrupted in *parg1* mutants [131]. Apart from that, induction of *AtPARG1* expression was attenuated in *atm* and *atr* mutant plants and vice versa [108]. Similar to ATM, ATR is an initiator of various DNA damage repair pathways [120]. Hence, PARGs, like PARPs, appear to act as a switch between different DNA repair pathways in planta. Yet, their exact function is still ambiguous. 

#### 3.3.4. Similar to Their Human Counterparts, Plant PARPs Are Capable of Chromosome Modification

In line with a role in plant DNA damage response, PARPs have a poly(ADP-ribosyl)ating activity on chromosomal proteins. AtPARP1 and AtPARP2 associate with chromosomes in dividing cells via their N-terminal domains [132]. Thereby, both proteins co-localize and probably compete for suggested heterochromatin association sites. Histones H1, H2A, and H2B in wheat and tobacco and histones H1.1 and H1.3 in Arabidopsis were targeted by poly(ADP-ribosyl)ation, putatively creating a chromatin structure more accessible to RNA polymerase II, as found for human PARP1 [86,87,101]. A potential transcriptional regulation by PARP proteins is indicated by the interaction of AtPARP1 with DIP1 and DIP2, two proteins homologous to the transcriptional coactivator ALY, via its DNA-binding domain in vitro and in yeast [133]. 

Expression of *AtPARP1* and *AtPARP2*, but not *AtPARP3*, is increased in response to telomerase dysfunction [97,134]. However, in contrast to their human counterparts, AtPARP1 and AtPARP2 do not stimulate telomerase activity. Apart from this, neither *Arabidopsis* PARP is involved in telomere end protection and telomere length protection in seedlings and flowers [97]. In contrast, HvPARP3 was suggested to be involved in telomere length maintenance in barley seedlings [114]. This apparent discrepancy calls for a systematic study of telomere-related roles of PARPs in a greater diversity of plant species.

### 3.4. Plant PARPs Play Diverse Roles in Cell Death, Development, and Metabolism

Mammalian PARP proteins are regulators of various facets of cell death (see Section 2.2). Comparable functions have also been found for plant PARPs [135,136]. Thus, treatment with the PARP inhibitors, 3AB and nicotinamide, blocked heat shock- and H_2_O_2_-induced PCD in cultured tobacco and soybean cells, whereby the PARP inhibitor-mediated protection from H_2_O_2_-induced PCD was most effective during the initial phase of H_2_O_2_ treatment [135,136]. At that point, a sharp drop in NAD^+^ levels in the soybean cells indicated the onset of PARP activity. Interestingly, overexpression of *AtPARP2* in soybean cell culture resulted in reduced cell death at low concentrations of H_2_O_2_ (mild oxidative stress), but a dramatically increased cell death at high H_2_O_2_ concentrations (severe oxidative stress). In addition, *AtPARP2* expression reduced the amount of nicked DNA under both mild and severe oxidative stress [135]. Additionally, cleavage of AtPARP proteins by Caspase-3, a central component of programmed cell death, was demonstrated in tobacco cells directly after PCD-inducing heat shock treatment [136]. These findings suggest that plant PARPs fulfill similar functions as their mammalian counterparts: They act as a switch between DNA damage repair under mild stress conditions and PCD under severe stress conditions. 

Apart from DNA damage and PCD, PARP proteins and poly(ADP-ribosyl)ation have been proposed to play an important role in plant development [137]. For example, the formation of tracheary elements in artichoke cell cultures, artichoke tubers, and pea root explants was inhibited by addition of the PARP inhibitor, 3AB [138]. In addition to this, *AtPARP1* and *AtPARP2* expression and their activity increased in *Arabidopsis* cell cultures during the exponential growth phase [105]. This increase in expression and activity was temporally linked to an increase in marker gene expression for S to G2 phase transition in the cell cycle. Simultaneously, there was a correlation between the increase in PARP activity and an increase in the glutathione pool during exponential growth of the cell culture [105]. Hence, PARP activity is linked to cell cycle progression and redox regulation, suggesting a regulatory function of AtPARPs in plant development. In line with this, seed germination was altered in *parp1*, *parp2*, and *parp3* mutant plants. Under non-stressed conditions, *parp3* plants germinated faster than the wild type, while *parp1* and *parp2* exhibited slower and partially reduced germination rates [93]. 

In addition to germination, plant growth regulation appears to involve poly(ADP-ribosyl)ation, since the PARP inhibitor 3MB has been shown to improve *Arabidopsis* growth under non-stressed conditions [106,139]. Enhanced growth by 3MB was attributed to higher leaf cell numbers due to a shortened cell division cycle, resulting in an increased overall leaf size [139]. Moreover, 3MB treatment altered gene expression in Arabidopsis plants under unstressed conditions, affecting components of plant responses to external and internal stimuli and abiotic stress response, circadian rhythm, plant growth, energy metabolism and photosynthesis, and primary and secondary metabolism [106,131,139]. For the PARP inhibitor 3AB, contrasting effects have been reported, depending on the 3AB concentration used. Biomass of Arabidopsis plants was reduced upon treatment with 2.5 or 5 mM 3AB [97,131,140], whereas 1 mM 3AB promoted plant biomass and root system development, resulting in more lateral roots, formation of secondary order lateral roots, increased lateral root length, and increased primary root length compared to the control plants [116]. Similar, although weaker, effects were observed for another PARP inhibitor, 6(5H)-phenanthridinone. Similar to pharmacological PARP inhibition, the absence of AtPARP1 and AtPARP2 in *parp1 parp2* double mutant plants led to the formation of a larger root system compared to the wild type [116], but this phenotype was weaker than that caused by the inhibitors. In contrast, in a recent study by our lab, knockout mutant lines of all *Arabidopsis PARP* genes, including all double mutant combinations and a *parp1 parp2 parp3* triple mutant, did not display obvious developmental alterations under non-stress growth conditions [47]. This is consistent with findings obtained by other authors [92,108,116]. Hence, PARPs seem to have an effect on plant development only under specific conditions the nature of which needs yet to be defined. Furthermore, the stronger and more consistent effects or PARP inhibitors, as compared to genetic knockout, points to off-target effects of those substances. Further work is required to elucidate the specific role of PARPs in plant development.

### 3.5. Poly(ADP-Ribosyl)ation and Plant Responses to Abiotic Stress

PARP proteins of plants have been associated with responses to abiotic stress. In some studies, the expression of *AtPARP* and *AtPARG* genes was altered in response to abiotic stresses [95,104], albeit numerous other studies failed to detect transcriptional responses to drought, osmotic, or salt stress [47,141,142,143,144,145,146,147]. Knockdown of *PARP1* or *PARP2* in oilseed rape and *Arabidopsis* by *PARP* hairpin constructs enhances the tolerance to desiccation, short-term paraquat treatment, and high light, associated with a reduced PARP activity under stress [99]. Accordingly, high energy consumption, which is a response to PARP activation following severe stress in mammalian cells, was prevented in the knockdown lines. Energy homeostasis and normal levels of mitochondrial respiration were maintained, and ROS production was kept low [99]. Hence, energy preservation under stress was suggested as a cause of the enhanced abiotic stress tolerance of *PARP* knockdown lines. Additional explanations were provided by a transcriptomic study on plants with reduced *AtPARP1* expression [107]. Under “high light” stress (250–300 µmol m^−2^ sec^−1^), *AtPARP1* knockdown led to an attenuated expression of temperature-responsive and oxidative stress-dependent genes. Furthermore, genes involved in cellular transport and energy metabolism were repressed, again supporting the hypothesis that reduced PARP activity enhanced stress tolerance by reducing oxidative stress and preserving energy homeostasis. In contrast, genes responsive to abscisic acid (ABA), dehydration, and cold were hyper-induced by *AtPARP1* knockdown under high light stress [107]. Simultaneously, ABA levels were observed to be higher in the plants exhibiting reduced *AtPARP1* expression. These findings pointed to a role of AtPARP1 as a negative transcriptional regulator of plant stress responses in an ABA-dependent way. This provoked an additional hypothesis to explain the enhanced abiotic stress tolerance by reduced PARP activity, linking the increased NAD^+^ levels to the increased ABA levels and the increased expression of ABA-responsive genes via cyclic ADP-ribose (cADPR) [107]. This molecule is synthesized by an unknown ADP-ribose cyclase using NAD^+^ as a substrate, and it is involved in ABA-dependent stress signaling, as well as in the regulation of the circadian clock [109,148,149,150]. cADPR triggers the release of Ca^2+^ from internal stores, which induces ABA production and hence ABA-responsive gene expression. It was hypothesized that the preservation of the NAD^+^ pool by a reduction in *AtPARP1* expression promotes cADPR synthesis, thereby conferring enhanced stress tolerance. Albeit the synthesis of cADPR, its activity as Ca^2+^-releasing second messenger, and its involvement in ABA and nitric oxide signaling have individually been demonstrated in plants [150,151,152,153,154], the underlying molecular mechanisms remain largely obscure since neither homologs of animal ADP-ribose cyclases nor those of animal cADPR-activated Ca^2+^ channels (e.g., ryanodine receptors) are present in higher plants [155]. The testing of this hypothesis therefore poses a formidable task.

The notion of plant PARPs as important negative factors of abiotic stress tolerance has been challenged by a recently reported study, wherein growth of *Arabidopsis* single, double, and triple knockout mutants for all three *AtPARP* genes was unchanged from the wild type when exposed to desiccation, salt, osmotic, or oxidative stress [47]. These opposing results lead to the conclusion that the enhanced stress tolerance phenotype is conditional. Conditional phenotypes occur frequently in loss-of-function mutants, since plants have evolved many adaptive traits that allow them to cope with changes in their environment [156]. Factors accounting for conditional phenotypes can be light, temperature, and nutritional status, and interactions among them are possible [156]. Accordingly, a large number of conditional phenotypes have been described in loss-of-function mutants which affect all aspects of plant development [157]. For instance, the conditional root expansion mutant *quill* shows similar root growth as the wild type on 0.5% sucrose, but dramatically reduced root growth on 4.5% sucrose medium. Under these conditions, root growth of wild type plants is even pronounced [158]. Similarly, the *petit1* mutant shows reduced hypocotyl elongation on sucrose-containing medium, but not on sucrose-free medium [159]. The photoperiod-insensitive early-flowering 3 mutant *elf3* shows rhythmic leaf movement in the dark and under several light/dark regimes, but not under constant light [160]. Not only the light regime, but also the light intensity, can affect plant phenotypes. Consequently, *vad1* mutants show hypersensitive response-induced lesions under high, but not under low, light intensities [161]. In contrast, plants lacking the Myeloblastosis (MYB) domain-containing proteins MYB33 and MYB65 exhibit male sterility specifically under low light conditions [162]. These examples illustrate the wide range of conditional phenotypes identified so far in plant research using loss-of-function mutant lines.

Compared to genetic PARP inhibition, pharmacological PARP inhibition has been widely used as a tool to elucidate the role of PARP proteins in plant stress tolerance [99,106,122,139,163]. Energy homeostasis in plants was improved by 3MB-mediated PARP inhibition. While the expression of genes related to photosynthesis, the effective photosynthetic quantum yield, and the electron transport rate were induced, low-energy-status marker genes were unaltered in *Arabidopsis* plants grown in unstressed conditions [139], and NAD^+^ content was increased under both unstressed and oxidative stress conditions [106]. Notably, 3MB treatment de-regulated gene expression of components of the phenylpropanoid pathway under unstressed and oxidative stress conditions [106,139]. The abundance of metabolites derived from the phenylpropanoid pathway, such as flavonols and lignins, decreased upon 3MB treatment [139]. These findings can explain reduced leaf pigmentation, reduced anthocyanin accumulation, and enhanced plant growth observed upon 3MB treatment under paraquat-induced oxidative stress [106]. Reduction of anthocyanin accumulation was not specific to 3MB, as the PARP inhibitors 3-methylbenzamide and 3-aminophthalhydrazide acted similarly. Additionally, concentrations of other stress-related metabolites, such as galactinol or myo-inositol, were reduced by the inhibitors. Apart from its interference in oxidative stress responses, 3MB enhanced plant growth in response to salt, heat, and high-light stress [106]. Additionally, 3MB and nicotinamide enhanced the tolerance of *Brassica napus* hypocotyl explants to oxidative stress elicited by acetyl salicylic acid [99]. In another study, the PARP inhibitor 4-amino-1,8-naphthalamide enhanced growth rates of *Lemna minor* facing osmotic stress by polyethylene glycol treatment (−0.3 MPa) and tolerance of an Arabidopsis cell culture to H_2_O_2_ [163]. In contrast to the finding that the pharmacological inhibition of PARP reduced plant sensitivity to paraquat treatment [99], other authors found that 3AB treatment of Arabidopsis seedlings enhanced the sensitivity to long-term paraquat treatment [122]. This discrepancy might be explained by different levels of stress and the resulting extent of PARP activation.

### 3.6. Poly(ADP-Ribosyl)ation and Plant Responses to Biotic Attack

Pathogen-associated molecular patterns (PAMPs) are structural or functional units of a microbe that are recognized by the plant immune system and elicit defense responses. The N-terminally conserved 22 amino acids of flagellin (flg22) from *Pseudomonas* bacteria and the N-terminal 18 amino acids of EF-Tu (elf18) from *Escherichia coli* are such PAMPs. They elicit immune responses such as oxidative burst, elevation of cytosolic free Ca^2+^, cell wall reinforcements by callose and lignin, and transcriptional induction of defense genes [164,165,166]. The PARP inhibitors 3AB and 6-(5H)-phenanthridinone block flg22- and elf18-induced callose deposition in *Arabidopsis* seedlings [47,102,130,140], albeit transcript and protein induction of the callose synthase gene are not affected [102]. This effect can be bypassed by salicylic acid [130]. No alterations upon PARP inhibitor treatment were found in early PAMP responses, such as ROS burst, and in wounding-induced callose deposition [102,130]. In contrast to 3AB and 6(5H)-phenanthridinone, the potent PARP inhibitors PJ-34, INH2BP, and 4ANI did not block PAMP-induced callose deposition [47,102], which may be explained by off-target effects of the drugs.

A number of other pharmacological studies have assigned PARPs a role as regulators of pathogen responses. For example, the PARP inhibitor 3MB, which is structurally very similar to 3AB, has been shown to negatively influence the phenylpropanoid pathway [106,139]. Similar observations were also made for 3AB, which inhibited phenylalanine ammonium lyase, a component of the phenylpropanoid pathway [167]. Likewise, elf18-induced guaiacyl-lignin accumulation was blocked upon 3AB treatment [130]. Transcriptomic analyses further revealed that pharmacological PARP inhibition by 3AB de-regulated PAMP-induced transcriptional responses in Arabidopsis [131].

Contrary to pharmacological PARP inhibition by 3AB and 6-(5H)-phenanthridinone, genetic abolishment of *AtPARP1* or *AtPARP2* in T-DNA single knockout lines did not alter flg22-induced callose deposition, whereas in two studies *parp1 parp2* double mutant lines exhibited enhanced or reduced callose deposition, depending on the plant age and/or flg22 concentration applied [92,168]: The transfer of five-day-old seedlings to liquid medium containing 1 µM flg22 enhanced callose deposition (but not the ROS burst) [92], while infiltration of leaves of four-week-old plants with 0.5 µM flg22 reduced this response [168]. Nevertheless, *parp2* mutant plants and *parp1 parp2* double mutant plants displayed enhanced sensitivity towards the *Pst* strain DC3000 [92,101]. This strain also induced an activation of PARP in Arabidopsis plants, as demonstrated by reduced cellular NAD^+^ levels and increased leaf poly(ADP-ribose) content [130]. Taken together, these findings point to a role of PARP proteins, particularly PARP2, as regulatory components of the basal immune response. However, in two independent recent studies, flg22-induced callose deposition was not altered in *parp1 parp2 parp3* triple knockout seedlings [47,102]. In the two studies, the plants differed in their developmental stage and their growth conditions at the time of flg22 application. Hence, these contrasting findings further support the above-raised notions of a conditionality of the *parp* mutant phenotypes and of off-target effects of PARP inhibitors, which remain to be tested experimentally.

A new actor in PARP-mediated plant immunity has been identified recently. Mutant plants lacking the AtPARP2-poly(ADP-ribosyl)ated protein DWADLE (DDL) exhibited an exacerbated sensitivity to *Pst* DC3000, *P. syringae pv. maculicola*, non-pathogenic *Pst* DC3000 hrcC (a type III secretion mutant of *Pst* DC3000), and non-adaptive pathogen *P. syringae pv. phaseolicola* [168]. Similarly, *ddl* mutants showed reduced callose deposition in response to flg22 and *Pst* DC3000 hrcC. In contrast to pharmacological PARP inhibition, early PAMP-induced gene expression was reduced in *parp1 parp2* mutants, but not in *ddl* [101,168]; late transcriptional responses to PAMP treatment were reduced in both *parp1 parp2* and *ddl* [168]. These partially overlapping phenotypes of *parp1 parp2* and *ddl* further indicated an interaction of these proteins in response to PAMPs. In fact, an interaction of AtPARP2 and DDL was confirmed by immunoprecipitation and bimolecular fluorescence complementation assays, and found to be enhanced by flg22. The direct interaction of both proteins was markedly stronger upon poly(ADP-ribosyl)ation. In addition to AtPARP2, AtPARP1 was also shown to poly(ADP-ribosyl)ate DDL, but to a lesser extent than AtPARP2. A DDL protein lacking its poly(ADP-ribosyl)ation sites was unable to complement the susceptibility to PAMP treatment, indicating that poly(ADP-ribosyl)ation of DDL is essential for proper plant immune response. As DDL enhances late PAMP-induced gene expression, and as it was suggested to interact with histone acetyltransferases in *Arabidopsis*, Feng and colleagues concluded that DDL is involved in poly(ADP-ribosyl)ation-mediated chromatin remodeling to allow access to target gene promoters during plant immunity [168].

Since PARGs antagonize PARP action, *parg* mutants should also display altered responses to biotic attack. Accordingly, *parg1* but not *parg2* mutants displayed a more severe growth inhibition upon elf18 treatment accompanied by an enhanced pigment accumulation [130]. Mature *parg1* plants also displayed enhanced flg22-induced callose deposition and increased expression of flg22-regulated genes [101]. Similar to *parp* mutant plants and pharmacological PARP inhibition, the early PAMP-induced ROS production was not altered in the *parg1* mutant plants. Nevertheless, this mutant displayed transcriptional changes in defense gene expression [131]. These changes could not be assigned to specific pathogen-induced signaling pathways, leading to the conclusion that AtPARG1 acts as a regulatory element at response pathway junctures [131]. Apart from this, the onset of symptoms of *B. cinerea* infection was accelerated in both *parg1* and *parg2* mutant plants [130]. Hence, particularly AtPARG1 can be described as a positive regulator of biotic stress responses. Against this background, it is surprising that *AtPARG2* expression was up-regulated in response to *Pst* and *Botrytis cinerea* infection and flg22 treatment, while *AtPARG1* was only transiently induced by treatment with flg22 and elf18 [92,130,140]. This discrepancy needs to be elucidated. 

### 3.7. Non-Canonical PARP Domain Proteins Act in Plant Stress Responses

In addition to the canonical PARP proteins, members of the SRO (Similar to RCD One) family possess a catalytic PARP domain, albeit with an unusual catalytic triad motif [169,170]. Therefore, SRO proteins can be regarded as part of the plant PARP family sensu lato. SRO genes are present in all sequenced land plant genomes, with considerable variation in composition between the sequenced plant species [170,171,172]. In *Arabidopsis*, the family comprises the proteins Radical-induced Cell Death1 (RCD1) and its homologues SRO1 through SRO5 [170,173]. In contrast to the canonical PARP proteins, the close homologues RCD1 and SRO1 contain a central catalytic PARP domain which is flanked by an N-terminal WWE domain and a C-terminal RST domain, while SRO2 through SRO5 lack the WWE domain (Figure 2) [169,174]. In in vitro assays, RCD1 is not enzymatically active [170], whereas, intriguingly, PARP activity has been demonstrated in vitro and in vivo for a homologous protein from wheat [175].

RCD1 is the first recognized member of this protein family. It was initially identified during screenings for ozone sensitivity in *Arabidopsis* and oxidative stress regulators in yeast [173,176]. Accordingly, *Arabidopsis rcd1* mutant plants were hypersensitive to ozone treatment and the resulting apoplastically-produced ROS [169,176]. In contrast, *rcd1* plants are resistant to ROS formed in the chloroplasts upon paraquat treatment [169,177]. *RCD1* is expressed ubiquitously and constitutively in Arabidopsis plants [174,178]. It localizes to the nucleus [179,180], but upon salt and oxidative stress, RCD1 is also localized in the cytoplasm [180]. RCD1 has been shown to be involved in responses to salt stress, in pathogen defense, in the regulation of stomatal conductance, in UV-B responses, in temperature responses, in PCD, in redox regulation, and in ROS and plant hormone signaling [169,177,180,181,182,183,184,185,186,187,188,189,190]. Interestingly, RCD1 is the target of an effector protein of the oomycete pathogen *Hyaloperonospora arabidopsis*, and binding of this effector suppresses the activation of salicylic acid-induced defense genes and alters light responses [184]. RCD1 is phosphorylated, likely by interacting with Mut9-like protein kinases that also phosphorylate photoreceptors. This may present a link of photoresponse and salicylic acid signaling [184]. *Rcd1* mutant plants show severe developmental defects, such as stunted growth, altered rosette and leaf morphology, early flowering time, and a high number of differentially regulated genes [169,174,178]. The RCD1 protein interacts physically with the Na^+^/H^+^ antiporter SOS1 and in particular with transcription factors from various families, such as AP2/ERF, NAC (NAM, ATAF1 and -2, and CUC2), MYB, and basic helix–loop–helix [173,174,180,191]. The most prominent transcription factor RCD1 interacts with is the AP2/ERF family protein DREB2A [173,174,192], which is a transcriptional regulator of genes involved in the responses to various stresses, such as drought, salinity, and heat, and which is also involved in plant senescence [192,193]. This interaction is mediated by the approximately 70 amino acids long, helically-structured C-terminal RST domain [174,190,194,195]. The term “RST” is named after the three proteins carrying this domain: RCD1, SRO1, and TAF4 [194]. In DREB2A, binding to RCD1 is conferred by the RCD1-interacting motif (RIM), albeit the RIM sequence is not sufficient for this interaction [192]. In other transcription factors interacting with RCD1, this motif is absent, and no common motif sequence has been identified conferring the interactions [192,196]. Instead, interaction between RCD1 and its transcription factor partners is mediated by intrinsically disordered regions [197]. Accordingly, short linear motifs of disordered transcription factor regions were found to be sufficient for RCD1 interactions [196]. In summary, the PARP domain protein RCD1 appears to be a central hub in plant stress and pathogen responses, ROS homeostasis, plant hormone signaling, and PCD. 

The PARP domain protein SRO1 shares 76% similarity with RCD1. Both proteins were identified to be paralogs likely arising from a gene duplication [178]. They both localize to the nucleus and are both expressed ubiquitously throughout the plant [174,178,198], whereby expression levels of *SRO1* are generally lower than those of *RCD1* [178]. In contrast to *rcd1* mutant plants, *sro1* plants did not exhibit sensitivity to ozone and salt stress, and grew normally under unstressed conditions [174,178]. Similar to RCD1, SRO1 interacted with transcription factors, but only with a subset of those interacting with RCD1 [174]. Double mutants lacking both RCD1 and SRO1 showed detrimental developmental defects [174,178,198]. Collectively, RCD1 and SRO1 appear to be unequally redundant proteins [174].

Another member of the SRO family is SRO5. In contrast to *RCD1* and *SRO1*, which show hardly any changes in their expression upon stress, *SRO5* transcript levels changed upon salinity, ozone, light, wounding, anoxia, and bacterial elicitors [170,199]. Additionally, SRO5 was shown to be involved in the response to salt and oxidative stress in a very peculiar way [200]: upon salt stress, *SRO5* transcripts form siRNAs with a gene overlapping in antisense orientation, *P5CDH* (1-pyrroline-5-carboxylate dehydrogenase), thus regulating it at the post-transcriptional level. Surprisingly, expression of the *P5CDH* gene was not enhanced in *sro5* mutant plants [170,199]. Apart from this, *SRO5* and *P5CDH* did not overlap in *Arabidopsis lyrata*, grapevine, and poplar [170]. It was therefore proposed that the regulation of *P5CDH* is not a primary function of the *SRO5* gene [170,199]. SRO5 itself is apparently involved in ROS regulation [200]. Similar to RCD1 and SRO1, SRO5 localized to the nucleus and interacted with transcription factors, including DREB2A [170], providing an alternative explanation for SRO5 action in planta. Similar to *SRO5*, *SRO2* and *SRO3* showed changes in their transcript levels in response to light, salt and ozone [170].

The central position of the SRO family in signaling and stress responses also holds true for monocots. The modulation of ROS homeostasis and a positive role in abiotic stress resistance have been demonstrated for a wheat homolog of RCD1/SRO1, Ta-SRO1 [175]. An SRO protein in rice, OsSRO1c, which is a target of a NAC transcription factor, is also involved in the resistance to oxidative stress, stomatal regulation, and resistance to drought and cold [171,201]. Like *Arabidopsis* RCD1 and SRO1, this rice protein interacts with DREB and NAC transcription factors [201]. Interaction with a NAC transcription factor was also shown for a barley homolog, HvRCD1 [191]. Another OsSRO family member, OsSRO1a, interacts with an RNA-binding domain protein, OsRBD1 [202]. Co-expression of both proteins in yeast resulted in improved growth under abiotic stress conditions, but a decreased tolerance to oxidative stress [202]. Recently, six SRO proteins have been identified in maize [203,204]. The promoter regions of the genes encoding these proteins contain stress-related and hormonal signaling-related elements. Accordingly, expression of *ZmSRO* genes was differentially affected by stress and hormone treatments [203,204].

Even though research on SRO proteins has mostly focused on *Arabidopsis* and monocotyledonous crop species, members of the family have recently been described in other dicotyledonous species. The tomato protein Sl-SROl1, most similar to AtSRO5, improved salt tolerance when expressed in *Arabidopsis* [199], and the RCD1 protein from lily interacted with the DREB2 transcription factor LlDREB2B, similar to its *Arabidopsis* ortholog [205].

### 3.8. Potential Off-Target Effects of PARP Inhibitors

In addition to their function in DNA damage responses (see Section 3.3), plant PARPs have been suggested before to be modifiers of plant development, robust negative regulators of plant abiotic stress responses and as positive factors of PAMP-triggered immunity (see Section 3.4, Section 3.5 and Section 3.6). This is largely based on studies employing pharmacological PARP inhibition, which appears to modify plant responses to stress, and also plant growth and development under unstressed conditions, more frequently and consistently than genetic *PARP* knockout [47,99,102,106,139,163]. This provokes the idea that pharmacological PARP inhibitors do not only affect the activity of canonical PARPs, but also have off-target effects in planta. In this respect, it is of note that many PARP inhibitors act unspecifically against diverse members of the PARP and tankyrase family in humans [206]. In Arabidopsis, the notion of promiscous PARP inhibitor activity is supported by the finding that protein poly(ADP-ribosyl)ation was not abolished in a *parp* triple mutant [47], which may be explained by other enzymes also possessing this activity. Proteins belonging to the SRO family may represent such potential alternative inhibitor targets since they contain a catalytic PARP domain. In silico analysis revealed that the PARP inhibitors 3AB and 6-(5H)-phenanthridinone may bind to this domain [47], whereas a recent study indicated that 6-(5H)-phenanthridinone does not bind to the catalytic PARP domain of RCD1 in the way it binds to human PARP1 [184]. Nevertheless, PARP inhibitors may have a disruptive role in RCD1 function and a yet undefined interaction with the catalytic PARP domain is still conceivable. The unequally redundant proteins, RCD1 and SRO1, as well as the SRO5 protein, have been reported repeatedly to be important actors in plant stress responses. This complex involvement of SRO proteins in stress responses matches the commonly observed changes in stress responses by pharmacological PARP inhibitors. 

The notion that PARP inhibitors have off-target effects is further supported by a recent transcriptomic study in which, under standard growth conditions, a treatment with the established PARP inhibitors 3AB and 3MB altered the expression of 228 and 3935 genes, respectively [131]. This difference of one order of magnitude makes it highly unlikely that the effects are caused by the inhibition of canonical PARP proteins alone. 

Due to the potential off-target effects, the use of pharmacological PARP inhibitors to infer PARP function in plants should be reconsidered. As a first step to identify additional targets of those drugs, pull-down experiments using the pharmacological “PARP inhibitors” as bait may be a suitable approach.

## 4. PARPs Under Stress—Concluding Remarks and Future Perspectives

In their natural habitat, plants are under the constant threat of biotic and abiotic stressors. Global warming is proposed to increase these threats, endangering future food security. The inhibition of PARPs by genetic or pharmacological means has been suggested as a promising approach to sustain crop yields and hence food security, since PARP inhibition has been found to improve plant performance under abiotic stress. However, the positive effects of PARP inhibition are not as robust as initially suggested, since it has become apparent that the stress-related phenotypes of *parp* mutants are of a conditional nature. This now calls for an extensive systematic analysis of growth conditions and plant developmental stages to identify the circumstances under which PARP modification causes an alteration of stress responses. 

The currently available data therefore suggest that inhibition of canonical PARPs may not be a way forward to stabilize food security. Moreover, PARP inhibition has been shown to exert negative effects on pathogen resistance mechanisms. However, simultaneous or sequential occurrence of abiotic and biotic stress elicits tailored physiological and transcriptional responses that differ from those to single stressors. Accordingly, abiotic and biotic stressors have been reported numerously to have either additive effects, i.e., increasing plant stress level and symptoms, or antagonizing effects, i.e., increasing resistance to one of the stressors [207,208,209]. For their general roles in genome stability and stress responses, canonical PARPs and SROs may be determinants in the coordination of plant responses to multiple stresses. To test this idea, the response to the combined appearance of abiotic and biotic stressors ought to be studied in plants with altered genetic setup of *PARP*s/*SRO*s or pharmacologically-altered PARP activity.

In this respect, it is an important future task to experimentally determine the potential additional targets of PARP inhibitors, as this may allow the design of specific inhibitors targeting those proteins. They may be of use in future crop improvement strategies, as the beneficial activities of canonical PARPs would not be affected. Finally, to better understand the mechanistic role of PARP domain proteins in stress responses, the identity of poly(ADP-ribosyl)ated proteins and poly(ADP-ribose)-binding proteins needs to be elucidated. 

## Figures and Tables

**Table 1:** Previously used and suggested nomenclature for *AtPARP1* and *AtPARP2* of *Arabidopsis thaliana*.

Reference	At2g31320	At4g02390
**Suggested Nomenclature**	**AtPARP1**	**AtPARP2**
Boltz et al. (2014), PLoS One 9: e88872 [97]	AtPARP2	AtPARP1
Briggs and Bent (2011), Trends Plant Sci. 16: 372–380 [98]	AtPARP2	AtPARP1
Chen et al. (2018), Front. Plant Sci. 9: 1581 [94]	AtPARP1	AtPARP2
De Block et al. (2005), Plant J. 41: 95–106 [99]	AtPARP2	AtPARP1
Doucet-Chabeaud et al. (2001), Mol. Genet. Genom. 265: 954–963 [95]	AtPARP1	AtPARP2
Jia et al. (2013), Plant Mol. Biol. 82: 339–351 [100]	AtPARP1	AtPARP2
Feng et al. (2015), PLoS Genet. 11: e1004936 [101]	AtPARP1	AtPARP2
Keppler et al. (2018), Front Plant Sci. 9: 1907 [102]	AtPARP1	AtPARP2
Lamb et al. (2012), Cell Mol. Life Sci. 69: 175–189 [103]	AtPARP2	AtPARP1
Ogawa et al. (2009), Plant J. 57: 289–301 [104]	AtPARP1	AtPARP2
Pellny et al. (2009), Mol. Plant 2: 442–456 [105]	AtPARP1	AtPARP2
Pham et al. (2015), Plant Mol. Biol. 89: 319–338 [93]	AtPARP2	AtPARP1
Rissel et al. (2017), Front. Plant Sci. 8: 59 [47]	AtPARP1	AtPARP2
Rissel et al. (2017), Anal. Biochem. 527: 20–23 [96]	AtPARP1	AtPARP2
Schulz et al. (2012), PLoS One 7: e37287 [106]	AtPARP2	AtPARP1
Song et al. (2015), PLoS Genet. 11: e1005200 [92]	AtPARP1	AtPARP2
Vanderauwera et al. (2007), PNAS 104: 15150–15155 [107]	AtPARP2	AtPARP1
Zhang et al. (2015), Sci. Rep. 5:15892 [108]	AtPARP1	AtPARP2

## Abbreviations

3AB	3-aminobenzamide
ADP-ribose	Adenosine diphosphate ribose
ALY	Ally of AML-1 and LEF-1
4-ANI	4-amino-1,8-naphtalimde
AP2/ERF	APETALA2/ethylene response factor
APP	Arabidopsis thaliana homologue of PARP
ARH3	ADP-ribose hydrolase 3
ATM	Ataxia telangiectasia-mutated
ATP	Adenosine triphosphate
ATR	Ataxia telangiectasia and Rad3-related protein
BER	Base excision repair
BRCT	Breast cancer susceptibility gene 1 C-terminus
cADPR	Cyclic ADP-ribose
DIP	DNA-binding domain interacting protein
DREB	Dehydration-responsive element/C-repeat-binding proteins
DSB	DNA double strand break
elf18	N-terminal 18 amino acids of EF-Tu
flg22	N-terminally conserved 22 amino acids of flagellin
GUS	β-glucoronidase
HR	Homologous recombination
MacroD1	Macrodomain-containing protein D
3MB	3-methoxybenzamide
MMEJ	Micro-homology-mediated end joining
MMS21	Methyl methanesulfonate sensitivity gene 21
MMS	Methyl methanesulfonate
MRE11	Mitotic recombination 11
MYB15	Myeloblastosis transcription factor 15
NAC	NAM, ATAF1 and −2, and CUC2
NAD	Nicotinamide adenine dinucleotide
NAP	Non-classical poly(ADP-ribose)polymerase
NHEJ	Non-homologous end joining
NIC2	Nicotinamidase2
NTR	N-terminal region
PAMP	Pathogen-associated molecular pattern
PARG	Poly(ADP-ribose) glycohydrolase
PARP	Poly(ADP-ribose) polymerase
PCD	Programmed cell death
P5CDH	1-pyrroline-5-carboxylate dehydrogenase
Pst	Pseudomonas syringae pv. tomato
RCD1	Radical-induced cell death1
REV7	Protein putatively involved in translesion synthesis
RST domain	RCD1-SRO-TAF4 protein domain
SAP domain	Protein domain named after SAF-A/B, Acinus, and PIAS
SMC	Structural maintenance of chromosomes proteins
SOS1	Salt overly sensitive 1
SRO	Similar to Radical-induced cell death one
SUMO E3	Small ubiquitin-like modifier E3
SSB	DNA single strand break
SWI	Protein involved in involved in sister chromatid cohesion and chromosome organization
TARG1	Terminal ADP-ribose glycohydrolase 1
TEJ	Means “bright” in Sanskrit, name given to the PARG1 after its discovery as a regulator of circadian clock output
WGR domain	Protein domain named after the most conserved central motif of the domain
WWE domain	Protein domain named after three of its conserved residues
XRCC	X-ray repair cross-complementing protein
ZAP	Zinc-finger poly(ADP-ribose)polymerase
